# Effect of different cooking water on flavor characteristics of mutton soup

**DOI:** 10.1002/fsn3.2546

**Published:** 2021-09-07

**Authors:** Bing Zhao, Baoguo Sun, Shouwei Wang, Yuyu Zhang, Mingwu Zang, Wang Le, Hui Wang, Qianrong Wu

**Affiliations:** ^1^ Beijing Key Laboratory of Flavor Chemistry Beijing Technology and Business University (BTBU) Beijing China; ^2^ China Meat Research Centre Beijing China; ^3^ Beijing Academy of Food Sciences Beijing China

**Keywords:** aroma, meat soup, metal ions, taste, water

## Abstract

The mutton flavor is affected by cooking water significantly, and the flavor of mutton is delicious and widely loved by consumers through an extremely simple processing in northwest China, such as Inner Mongolia, Ningxia, and Xinjiang. The flavor shows obvious changes if get out of these areas even use the same raw meat, which may be caused by different cooking water. To determine whether and how the cooking water affect the mutton soup flavor, the elements in water, the flavor was studied by inductively coupled plasma mass spectrometry (ICP‐MS), amino acid analyzer, and thermal desorption (TDS)–gas chromatography–mass spectrometry (GC‐MS). Specifically, three water samples from different sources, Ningxia (NXW), Beijing (BJW), and ultrapure water from the laboratory (PUW), were used for cooking with Tan sheep's ribs to get different mutton soups. The inductively coupled plasma mass spectrometry (ICP‐MS) results showed that the elements and the concentration of solutes in different water sources were significantly different. The NXW batch had the highest Na, Mg, K, and Sr concentrations, and Na in NXW water reached to 50.60 mg/L, which existed as Na^+^, significantly (*p* < .05) higher than BJW (8.63 mg/L) and PUW, which were important to the flavor of mutton soup. The PUW batch had the highest content of free amino acids, and the content of glutamic acid (Glu) reached to 17.89 μg/mL. The NXW batch had the highest content of taste nucleotides, and the content of 5´‐IMP reached to 68.68 μg/ml. The volatiles of the three batches had significant differences, and only 40 volatiles were detected in all batches. Further flavor studies using electronic nose and electronic tongue showed significant differences in overall aroma and overall taste, especially bitterness, saltiness, and astringency. The results could provide a basis for improving the flavor quality for the mutton soup.

## Graphical abstract

In this study, different kinds of cooking water were used in mutton soup preparation to characteristics its effects. The types and concentrations of elements in different types of water showed significant differences. The overall aroma and overall taste had significant differences, especially in bitterness, saltiness, and astringency.

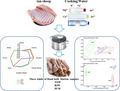

## 1 INTRODUCTION

Mutton, as one of the most important and popular meat product sources, has been widely accepted by consumers for its unique texture and flavor (Du et al., 2020; Saowakon et al., 2008). About 70% of consumers worldwide consider mutton as part of their daily diet and even as the main dish in several countries, especially in Asia, Europe, and America (Bueno et al., 2014). Moreover, some consumers, who do not like to eat mutton, have also changed their minds and gradually accept it, and some of them even become to enjoy it (Banskalieva et al., 2000; Zhan et al., 2017). The flavor, including aroma and taste, is the most important quality and attribute of mutton (Calkins & Hodgen, 2007; James & Calkins, 2008), and it is also the most important factor for mutton to be accepted by consumers (Mottram, 1998). Nevertheless, some consumers cannot accept the inherent flavor of mutton, which may be related to cultural traditions, social conditions and consumption habits, and so on (Gąsior & Wojtycza, 2016; Stanisz et al., 2009). In order to solve the problem, many efforts have been carried out, such as ameliorating species (Erasmus et al., 2017), feeding methods (Liu et al., 2019), slaughtering (Pophiwa et al., 2016), and processing (Tolentino et al., 2016), and satisfactory results have been achieved.

There are many methods to cook mutton to give the product different flavors, such as boiling, roasting, and frying. People in many countries and regions have the habit of drinking soup due to its flavor, nutrition, and benefits. The soup contains a variety of peptides, amino acids, and fatty acids, which can also increase satiety (Sun et al., 2011). Boiling is one of the popular cooking methods for mutton; the mutton and the soup are delicious and widely enjoyed. Mutton is a multi‐component complex system consisting of proteins, lipids, water, carbohydrates, inorganic, vitamins, and so on, which is the basis for the mutton flavor (Paulos et al., 2015; Whitfield & Mottram, 1992). During boiling, proteins, lipids, vitamins, and other substances are oxidized and degraded, and free amino acids, free fatty acids, peptides, inosinic acids, etc., are formed. These substances dissolve in water, giving the soup a favorable aroma and taste. In the soup, some substances are volatile aroma compounds, which primarily determine their aroma attribute. They are formed based on lipid oxidation and degradation, Maillard reaction, ribonucleotide degradation, amino acids and peptides degradation, and the interaction of lipid oxidation products with Maillard reaction products, as well as vitamin degradation (Farmer & Mottram, 1992; Madruga et al., 2010; Wood et al., 2004). Some substances are nonvolatile compounds, such as free amino acids, inosinic acids, and peptides, which could be perceived by the tongue. These ingredients are the basis for the flavor of mutton soup, resulting in a diversified system and forming a distinctive sensory profile (Mottram et al., 1982). However, the quality of the soup is unstable and seriously affects the sensory experience of the consumers.

The flavor of mutton soup is influenced by various factors, such as temperature, time, NaCl content, and raw meat. It has different effects on the formation of mutton soup flavor, and some satisfactory results had been achieved. The oxidation and degradation of proteins and lipids are the research hotspot, and it plays an important role in the formation of mutton soup flavor (Erasmus et al., 2017; Khan et al., 2015). The lean meat of mutton provides the basic meat flavor related to protein degradation and heterocyclic compound formation (Pearson et al., 1973). On the other hand, the unique flavor of mutton comes from the lipids, and the proportion of fatty acids in fat, especially unsaturated fatty acids, determined the flavor characteristics. 4‐methyloctanoic acid (MOA), 4‐ethyloctanoic acid (EOA), and 4‐methylnononic acid are typical branched‐chain fatty acids (BCFAs) and are considered to be the main contributors to the flavor, especially MOA (Wood et al., 2008). When the concentration of BCFAs reaches a certain level, the specific flavor of mutton will be perceived. However, the problem of unstable quality in mutton soup has not been completely resolved, and the mechanism of mutton soup flavor formation still remains to be elucidated.

Metal ions in cooking water can significantly affect the mutton and soup's flavor, Na^+^ can increase the solubility (Zhou & Yang, 2020) and taste of protein, K^+^ is the ion closest to the quality of meat and soup, but the bitterness and metallic taste limit the application (Zhang et al., 2016). Mg^2+^ can improve the gel properties within the appropriate concentration range, but the high concentrations will destroy the gel properties and show a metallic taste. Ca^2+^ can improve the meat masticatory and color but significantly reduce the water retention of meat (Choi et al., 2014; Horita et al., 2011). Metal ions can improve the dissolution of proteins in water and promote oxidation and hydrolysis of lipids and proteins to accelerate the formation and promotion of mutton soup flavor. Water contains various metal ions, such as Na^+^, K^+^, Mg^2+^, and Ca^2+^, and types and contents of water from different regions water show significant differences. Therefore, it is important to research the influence of the water from different regions on the soup's flavor characteristics.

Tan sheep are raised in Ningxia of China, with a unique flavor and smooth texture. Boiled mutton and soup made from Tan sheep are delicious and widely accepted by consumers. However, the flavor is changed once the tan sheep left Ningxia, which seriously affects the consumption of tan sheep.

The subjects of this study are tan sheep, and the water from Wuzhong district of Ningxia, Fengtai district of Beijing, and ultrapure water are used for cooking, focusing on the effect of different cooking water on mutton soup flavor (aroma and tastes), and the relationship among them is examined. Thus, through understanding the mechanisms of flavor formation influenced by metal ions, the mutton soup quality can be improved.

## 2 MATERIALS AND METHODS

### 2.1 Materials

The tan sheep was 3‐month age and obtained from Xinhai Food Co., Ltd. The cooking water was municipal water from Wuzhong district of Ningxia and Fengtai district of Beijing, and ultrapure water from the laboratory.

### 2.2 Processing of mutton soup

Tan sheep's ribs were divided into three batches and cooked with three different types of water. The batches were labeled as per the sources of cooking water: water from Ningxia (NXW), water from Beijing (BJW), and ultrapure water from the laboratory (PUW). During the sample preparation, the ribs were mixed with water 1:1.5 (w/w), and then, the mixture was heated by boiler with 2000 W for 10 min and 500 W for 50 min, respectively. Finally, the soup was collected and stored at −80℃ for analysis and completed in 2 weeks.

### 2.3 Determination of elements in water

Elements in three types of water were measured by inductively coupled plasma mass spectrometry (ICP‐MS, ICAP RQ, Thermo Fisher). Sodium, magnesium, aluminum, potassium, calcium, manganese, iron, copper, zinc, selenium, and strontium were characterized from three samples, respectively.

### 2.4 Determination of free amino acids

Free amino acids in the mutton soup were measured following the procedure by Wu et al. (2003) and Zhou et al. (2020) with minor modifications. First, the mutton soup was filtered with a medium‐speed filter paper, and the filtrate was collected. Then, three aliquots of 5 mL mutton soup filtrate samples mixed with 5  mL 5% sulfosalicylic acid solution, centrifuged at 10,000 g for 10 min at 4℃, and the supernatant was adjusted to pH 2.0 and through 0.22 μm nylon membrane filter. Then, the samples were subject to the automatic amino acid analyzer (L‐8900, Hitachi).

### 2.5 Determination of nucleotide

Nucleotides of mutton soup were measured following the procedure by Pu et al. with some modifications (Pu et al., 2020). The mutton soup was filtered with medium‐speed filter paper, and the filtrate was collected. Three aliquots of 10 mL mutton soup filtrate samples mixed with 10 mL 10% HClO_4_ solution, centrifuged at 10,000 g for 10 min at 4℃. Then, the precipitates were repeated with the same method. Both supernatants were mixed and filtered with medium‐speed filter paper, and the filtrate was adjusted to pH 5.4 with 1 mol/L NaOH. The final volume was adjusted to 50  mL with purified water and through a 0.45 μm nylon membrane filter. Of 10 μL aliquots were used for high‐performance liquid chromatography (HPLC). The contents of nucleotides in mutton soup samples were determined with HPLC (e2695, Waters) –DAD (2996, Waters). A C18 100 RP‐18 reversed‐phase column (SunFire C18, 4.6 mm×250 mm, 5 μm) was used for chromatographic separation. The elution program was isocratic elution with 5:95 (v/v) methanol: dipotassium hydrogen phosphate at a flow rate of 1.0 mL/min at 260 nm.

### 2.6 Determination of volatiles

Volatiles of the mutton soup were extracted by dynamic headspace sampling (DHS) and analyzed by thermal desorption (TDS3, Gerstel)–gas chromatography–mass spectrometry (GC‐MS, Trace1310‐TSQ8000, Thermo Fisher Scientific). Three aliquots of 20 mL of mutton soup were added 1  μL 0.816 μg/μL 2‐methyl‐3‐heptanone as internal standard and trapped in a Tenax TA tube (17.8 cm ×0.6 cm ×0.4 cm) which packed with Tenax TA (180 mg) at 50℃. High purity nitrogen was used as the carrier gas, and the flow rate was 50 mL/min. Then, Tenax TA tubes were fitted to TDS3, desorbed at 220℃ (heating rate 40℃/s), and cold focused onto a packed cold trap at −100℃ with Cold Inlet System (CIS 6, Gerstel). Volatiles was detected using TRACE 1310 GC with TSQ 8000 MS. ATG‐Wax MS column (30 m × 0.25 mm ID×0.25 μm, Thermo Fisher Scientific) was used for chromatographic separation. The temperature of GC injection port was 220℃, the GC temperature program employed by 3 min at 40℃, a ramp of 10℃/min to 180℃ and held for 5 min, and a ramp of 10℃/min to 220℃ and held for 10 min. High purity helium was used as the carrier gas, and the flow rate was 1.0 mL/min.

### 2.7 Analysis of electronic nose

An aliquot of 20 ml of mutton soup was transferred into a headspace bottle, balanced at 50℃ for 5 min, then 10 mL gas injected into the electronic nose (SuperNose‐14, ISENSO corporation, NYC). The acquisition time was 90 s, and each sample was tested five times. The data were analyzed by the software of Win muster for principal component analysis (PCA) and linear discriminant analysis (LDA).

### 2.8 Analysis of electronic tongue

The mutton soup was filtered with medium‐speed filter paper, and the filtrate was collected and then detected with INSENT Taste Analysis System (TS‐50000Z).

### 2.9 Statistical analysis

SPSS 17.0 was used for the majority of data processing. The one‐way analysis of variance (ANOVA) was used to determine the significant differences, and the*p* < .05 was considered as a significant level (Pu et al., 2021). Data were presented as means ±standard deviations (*SD*) of triplicate determinations. The correlation of parameters was evaluated by Pearson's correlation.

## 3 RESULTS AND DISCUSSION

### 3.1 Analysis of metal elements in water

The metal elements in water were important to plants, animals, and human beings. There were significant differences in metal elements in different water regions, which were mainly related to geographical conditions. A moderate amount of elements were beneficial, while excessive amounts were harmful. The metal elements in different water were shown in Table 1. There was almost no metal element in ultrapure, which was not listed in Table 1. There were significant differences in metal elements among NXW, BJW, and PUW (*p < .05*), especially in Na, Mg, Fe, Cu, Zn, and Sr. The metal elements exhibited different valence states in water and forming metal ions with different charges. Some metal elements only had one ionic valence, such as Na^+^, Mg^2+^, and K^+^, but some metal elements had several different ionic valences: the element Fe had Fe^2+^ and Fe^3+^, the element Mn had Mn^2+^ and Mn^3+^, and the element Se had Se^2‐^, Se^4+^ and Se^6+^. The types and concentrations of metal ions were also significant to meat processing and could significantly impact the quality of meat products.

**TABLE 1:** Content of metallic elements in different regions water

	Na	Mg	Al	K	Ca	Mn	Fe	Cu	Zn	Se	Sr
	(mg/L)	(mg/L)	(μg/L)	(mg/L)	(mg/L)	(μg/L)	(μg/L)	(μg/L)	(μg/L)	(μg/L)	(μg/L)
NXW	50.60 ± 0.85^a^	19.90 ± 0.21^a^	21.80 ± 0.25^b^	2.15 ± 0.14^a^	4.40 ± 0.14^b^	0.41 ± 0.05^b^	‐^c^	0.63 ± 0.05^b^	‐^d^	‐^e^	505.00 ± 5.36^a^
BJW	8.63 ± 0.12^b^	7.51 ± 0.14^b^	34.50 ± 0.65^a^	1.71 ± 0.12^b^	4.87 ± 0.11^a^	1.71 ± 0.12^a^	53.10 ± 0.32	28.10 ± 0.23^a^	111.00 ± 2.31	0.16 ± 0.02	173.00 ± 3.24^b^

Note

For the letters a and b, mean values (*n* = 3) with different letters within the same column are significantly different (*p < .05*).

‐ means not detected.

The quantitative limits of Fe were 0.9 μg/L; the quantitative limits of Zn were 0.8 μg/L; the quantitative limits of Se were 0.09 μg/L.

The metal ions of Na^+^, K^+^, Mg^2+^, and Ca^2+^ were commonly used in meat product processing and also could significantly affect the quality of meat products and soup. In NXW water, the element of Na reached 50.60 mg/L, which existed as Na^+^, significantly (*p < .05*) higher than BJW (8.63 mg/L) and PUW; the element of Mg reached 19.90 mg/L, which existed as Mg^2+^, significantly (*p < .05*) higher than BJW (7.63 mg/L) and PUW. In meat product processing, Na^+^ could affect the solubility of the myofibrillar protein, and low concentration of Na^+^ would reduce the strength of myofibrin protein gel (Haug et al., 2004; Sow & Yang, 2015); when the concentration of Na^+^ reach 0.5 mol/L, Na^+^ could promote protein gel formation and improve protein gel properties significantly (Chen et al., 2016) and the reason was that Na^+^ could change the ionic strength and result in reduction of a‐chain electrostatic bridges, consequently to shield the short range electrostatic interactions; Na^+^ could change hydrogen bond and interfere hydrophobic interaction (Choi & Regenstein, 2000). Na^+^ also could contribute to the formation of salty and umami flavor (Lou et al., 2017), due to Na^+^ may weak the adsorption of flavor compounds by various proteins and promote the release of flavor compounds(Ventanas et al., 2010). When the concentration of Na^+^ exceed 0.5 mol/L, myofibrin expanded and the ability of bind water is improved, which may be caused by the enhancement of electrostatic repulsion between myofilaments and the depolymerization of myosin filaments, the site where myofibrin binds to flavor is buried and loses bind ability(Xiong, 2005). K^+^ had similar physical and chemical properties to Na^+^ and could be used as an alternative for Na^+^, but excessive use of K^+^ would cause bitterness and metal odor. Mg^2+^ was a necessary macroelement in life activities and involved in multiple metabolisms. Mg^2+^ and Na^+^ have similar characteristics and could affect the gel properties (Barat et al., 2011).

Generally, Ca^2+^ had two sides to meat products, high levels of Ca^2+^could reduce protein extraction and strengthen protein–protein aggregation, while low concentrations of Ca^2+^ could improve the sausage quality under lower fat content (Kim et al., 2018; Wang et al., 2019). Ca^2+^ could facility the gelling of proteins and could bound volatile molecules; thus, the retention of flavor could be enhanced. Ca^2+^ can promote the polymerization of proteins in a certain concentration range, form the protein–metal ion aggregates, and further take shape protein gels with certain strength (Zhou & Yang, 2019). Ca^2+^ can coordinate with proteins at the negative positions between adjacent protein molecules to form ionic bonds, and the electrostatic repulsion and hydrophobic interaction between proteins may be changed to induce the intermolecular polymerization, (Navarra et al., 2014; Zhou & Yang, 2019). Ionic strength was another important factor for meat processing and played a key role in the formation and enhancement of texture due to their ability to dissolve myofibrillar protein and also could improve the flavor of meat products and soup (Han et al., 2019).

### 3.2 Analysis of free amino acids

Most amino acids showed a complex taste, such as sour, salty, umami, sweet, and bitter, while natural amino acids usually showed bitter taste related to the structure. The effect of cooking water on free amino acids in the mutton soup was summarized in Table 2. The level of the free amino acids in different batches of mutton soup showed significant differences (*p < .05*). The soup cooked with ultrapure water had the maximum content of total free amino acids, reached 831.19 μg/mL, and significantly higher (*p < .05*) than NXW and BJW batch. Glutamate, glycine, alanine, and aspartic acid were the main factors that determine the taste of food, collectively referred to as flavor amino acids, especially for Asp and Glu, which were important to the quality of the soup. The content of glutamate in pork soup (Rotola‐Pukkila et al., 2015) and chicken soup (Yang et al., 2018) was much higher than aspartic acid, which was similar to our results. Most of them had higher content in the PUW batch than other batches. All three batches had a low content of Asp, but the contents of Glu in three batches were higher and could show strong umami taste because of the low threshold value. In the PUW batch, the content of Glu reached 17.89 μg/mL and significantly higher (*p < .05*) than NXW batch and BJW batch. Ala also showed umami taste, and the content in the PUW batch reached 718.62 μg/mL. Gly was the only amino acid that showed umami taste in NXW batch instead of the PUW batch, reaching 7.54 μg/mL.

**TABLE 2:** Differences of free amino acids in different soup batches

Type	Taste characteristics	PUW content (μg/mL)	NXW content (μg/mL)	BJW content (μg/mL)
Asp	Sour/salty/umami	0.33 ± 0.01^c^	0.14 ± 0.00^a^	0.08 ± 0.00^b^
Thr	Bitter	8.05 ± 0.15^a^	7.94 ± 0.15^a^	4.47 ± 0.05^b^
Ser	Sweet/salty/umami	0.97 ± 0.02^c^	0.50 ± 0.01^a^	0.83 ± 0.01^b^
Glu	Sour/salty/umami	17.89 ± 0.34^c^	10.99 ± 0.21^a^	7.18 ± 0.07^b^
Gly	Sweet/ Salty/umami	6.21 ± 0.12^c^	7.54 ± 0.15^a^	4.34 ± 0.05^b^
Ala	Sweet/ salty/umami	718.62 ± 13.64^c^	550.76 ± 10.45^a^	465.70 ± 4.72^b^
Cys	Sweet/umami/salty	0.12 ± 0.00^c^	0.07 ± 0.00^a^	0.05 ± 0.00^b^
Val	Bitter	4.53 ± 0.09^b^	2.65 ± 0.05^a^	2.63 ± 0.03^a^
Met	Sweet/ salty/umami	1.91 ± 0.04^c^	0.93 ± 0.02^a^	0.71 ± 0.01^b^
Ile	Bitter	2.27 ± 0.04^c^	1.28 ± 0.03^a^	0.98 ± 0.01^b^
Leu	Bitter	5.77 ± 0.11^c^	3.23 ± 0.06^a^	2.55 ± 0.03^b^
Tyr	Bitter	2.22 ± 0.04^c^	1.04 ± 0.02^a^	0.91 ± 0.01^b^
Phe	Bitter	3.95 ± 0.08^b^	0.28 ± 0.01^a^	0.24 ± 0.00^a^
Lys	Bitter/sweet	1.58 ± 0.03^c^	0.46 ± 0.01^a^	0.32 ± 0.00^b^
His	Bitter/sweet	1.95 ± 0.04^c^	0.89 ± 0.02^a^	0.36 ± 0.00^b^
Arg	Bitter/sweet	8.78 ± 0.17^c^	4.49 ± 0.09^a^ '	2.75 ± 0.03^b^
Hypro	Sweet/bitter	19.86 ± 0.38^c^	14.25 ± 0.27^a^	5.58 ± 0.06^b^
Pro	Sweet/bitter	26.17 ± 0.50^c^	21.24 ± 0.41^a^	0.03 ± 0.00^b^
Total		831.19 ± 15.78^c^	628.65 ± 11.93^b^	499.71 ± 5.07^a^

Note

For the letters a‐c, mean values (*n* = 3) with different letters within the same row are significantly different (*p < .05*).

Some reasons may cause these different contents of free amino acids. First, different cooking water led to different heat‐degradation rates of protein, and protein in mutton was hydrolyzed to free amino acids during cooking. The muscle tissue structure was damaged, and the water‐soluble taste active compounds could transfer into soup from meat during stewing (Hou et al., 2018). There was almost no ion in ultrapure water, and the mutton had some ions, such as Na^+^, Ca^2+^, and Fe^2+^. The mutton is immersed in water, and the ions got into the water during cooking and reached a balance between water and mutton. And meanwhile, the content of free amino acids in mutton soup gradually increased, contained amino acids existed in natural, and formed by protein heat‐degradation. The ultrapure water had almost no ions and with a large concentration difference. As a result, a tremendous amount of free amino acid accompanied ions in mutton got into the soup. Therefore, the soup cooked by ultrapure water contained maximum content free amino acids. Second, Na^+^ was important for meat products and soup. An appropriate concentration of Na^+^ could promote protein dissolution in water and accelerate the protein degradation and free amino acids formation further. Ions such as K^+^ and Ca^2+^ played important roles in activating and inhibiting the activity of enzymes in meat, affected protein degradation and FAA release (Zhou et al., 2020). Some ions likely promote the recognition of proteins by proteases and generate more FAA (Wen et al., 2019). The NXW batch, which was cooked by Ningxia water, also had higher content amino acids because of the higher concentration of ions. It may form complexes with proteins, peptides, and other materials, affecting the free amino acids formation.

Moreover, the free amino acids may also reduce during processing and were related to the consumption, which contributed to the formation of flavor components, amines, and other substances (Sárraga et al., 1993). The content of protein in mutton was high, but it cannot provide a direct contribution to flavors, such as macromolecular myofibrillar protein and sarcoplasmic protein. Protein forms amino acids and peptides according to degradation reaction and indirectly contributed to flavor formation. These substances were not only the taste components but also the aroma precursors and formed aroma compounds through Maillard reaction and Strecker degradation. Maillard reaction was one of the extremely important flavor‐producing reactions and was necessary for ideal mutton flavor formation, which was mainly due to the interaction between amino acids and reducing sugars, and may cause the reduction of free amino acids, especially sulfur‐containing amino acids, such as methionine and cysteine (Buttery et al., 1977).

### 3.3 Analysis of nucleotides

Nucleotides are important in meat flavor perception, as they could provide umami taste characteristics. Nucleotides were consisting of purine bases or pyrimidine bases, ribose or deoxyribose, and phosphoric acid and had important physiological functions for life. Taste nucleotides were important for food processing and could significantly improve the food flavor when combined with L‐glutamate, which was a method to increase umami in meat processing. The effect of cooking water on nucleotides in the mutton soup was summarized in Table 3. Nucleotide levels of different batches showed significant differences (*p < .05*). 5'‐guanylic acid (5'‐GMP) and 5'‐hypoxanthine nucleotide (5'‐IMP) were the main nucleotides for umami. The soup cooked with Ningxia water had the highest contents and reached 8.72 and 68.68 μg/mL separately; adenylic acid (AMP) had a similar trend. The contents of 5'‐GMP and 5'‐IMP in the PUW soup, which was cooked with ultrapure water, had the lowest content, only 2.92 and 23.95 μg/mL, respectively. However, the inosine (I) in PUW batch had a higher content than that of the BJW batch and lower than the NXW batch. The content of hypoxanthine (HX) in the PUW batch was the highest and the lowest in the BJW batch.

**TABLE 3:** Differences of nucleotides in different batches of soups

	Taste characteristics	PUW (μg/mL)	NXW (μg/mL)	BJW (μg/mL)
5´‐GMP	Umami (+)	2.92 ± 0.05^c^	8.72 ± 0.16^a^	6.47 ± 0.23^b^
5´‐AMP	Umami/Sweet (+)	22.61 ± 1.18^a^	23.92 ± 1.09^a^	17.21 ± 0.87^b^
5´‐IMP	Umami (+)	23.95 ± 1.15^c^	68.68 ± 2.11^a^	51.60 ± 1.33^b^
I	Bitter (−)	63.76 ± 0.78^c^	72.36 ± 0.74^a^	46.00 ± 0.81^b^
HX	Bitter (−)	15.40 ± 0.33^c^	9.00 ± 0.67^a^	7.13 ± 0.27^b^

Note

For the letters a‐c, mean values (*n* = 3) with different letters within the same row are significantly different (*p* < .05).

The flavor of the mutton soup is mainly determined by taste nucleotides and the cooperative effect of taste nucleotides and flavor amino acids. 5'‐GMP and 5'‐IMP were the most important ingredients of mutton soup and demonstrated a more intense umami taste when combined with umami amino acids in the right proportion (Yamaguchi et al., 1971). 5ʹ‐GMP was a flavor enhancer that was stronger than MSG. 5ʹ‐GMP was a flavor enhancer which was stronger than MSG. The NXW batch had the highest 5'‐GMP and 5'‐IMP, indicating that the soup had a more umami taste. 5'‐AMP was formed by adenosine triphosphate (ATP) under enzymolysis and further formed IMP under dehydrogenation. 5ʹ‐AMP and 5ʹ‐IMP showed a synergistic effect that elicited the umami taste. Under the action of phosphokinase, part of IMP could form I and formed Hx under hydrolase. The ionic strength in Ningxia water was significantly higher than other batches, especially for Na^+^ and Mg^2+^. Appropriate ionic strength could improve the activity of the enzyme and further promote the release of taste nucleotide in water. There were two degradation pathways for ATP: the first was ATP →ADP → AMP →AdR → HxR →Hx, and the second was ATP →ADP → AMP →IMP → HxR →Hx. The first pathway was easier to accumulate 5'‐AMP, and the second one was to degrade 5'‐IMP with the AMP deaminase, and the deamination of AMP was also caused the increase of IMP. Therefore, the different contents of taste nucleotides in each batch may be related to the reaction pathway caused by ionic strength. According to the content of the taste nucleotides, the degradation pathway of ATP in mutton soup processing may be the second. High temperature and ionic strength could promote degradation of ATP and rapid accumulation of 5'‐IMP. This phenomenon was also found in pork broth (Yang et al., 2018) and chicken soup (Wang et al., 2012). The content of I in the PUW batch was higher than the BJW batch. Although the IMP was the lowest, the probable reason was that the IMP was instability in the environment of low ionic strength and formed I by degradation.

### 3.4 Analysis of volatiles

A total of 82 volatiles were identified in the mutton soup samples by DHS‐GC‐MS (Table 4). However, only 40 volatiles were detected in all batches of soup. The type and content of volatile compounds in different batches had significant differences. These findings indicated that the physicochemical and metabolic occurred during soup cooking. The content of volatile compounds in the PUW batch reached 104.45 μg/mL and much higher than the NXW and BJW batches, which only were 65.03 and 67.38 μg/mL. The aroma formation in the soup was very complex since the mutton was a multi‐component complex system and consisted of macroscopic (e.g., water, protein, lipids, carbohydrates, and inorganic) and trace components (e.g., vitamins, sugars, and nucleotides). The formation of soup aroma may include oxidation and thermal degradation of lipids, rib nucleotides, and thiamine, the interaction between sugars and amino acids (or peptides), as well as the pyrolysis of amino acids and peptides. In addition, the secondary reactions may occur between the products of these primary reactions and made further efforts to contribute to the development of mutton flavor (Xi et al., 2019).

**TABLE 4:** Differences of volatile compounds in different batches of soups

Compound name	CAS #	UPW μg/mL	NXW μg/mL	BJW μg/mL	RI value
Decane	124‐18‐5	2.55	1.24	1.57	1000
Undecane	1120‐21‐4	0.56	0.50	0.39	1099
Dodecane	112‐40‐3	5.15	5.12	5.16	1199
Tridecane	629‐50‐5	N.D	N.D	0.36	1298
Tetradecane	629‐59‐4	6.20	4.97	4.31	1398
Hexadecane	544‐76‐3	1.09	2.41	N.D	1598
Heptadecane	629‐78‐7	N.D	N.D	0.79	1699
Octadecane	593‐45‐3	1.16	0.75	1.07	1797
Nonadecane	629‐92‐5	4.49	0.94	2.15	1896
(Z)‐3‐Dodecene	7239‐23‐8	0.63	N.D	N.D	1214
Styrene	100‐42‐5	N.D	0.48	0.43	1257
1‐Nonene	124‐11‐8	N.D	0.19	N.D	1353
Longifolene	475‐20‐7	2.33	2.04	2.04	1558
D‐Limonene	5989‐27‐5	2.55	3.83	3.10	1195
Benzene	71‐43‐2	0.11	N.D	0.08	981
Ethylbenzene	100‐41‐4	1.67	0.57	0.78	1125
Propyl‐Benzene	103‐65‐1	0.20	0.16	0.26	1207
2‐Propenyl‐Benzene	300‐57‐2	N.D	0.12	N.D	1404
Butyl‐Benzene	104‐51‐8	N.D	N.D	0.11	1310
1‐Butanol	71‐36‐3	0.32	0.25	0.30	1143
1‐Pentanol	71‐41‐0	0.68	0.38	N.D	1265
1‐Hexanol	111‐27‐3	0.51	N.D	N.D	1360
2‐Ethyl‐1‐Hexanol	104‐76‐7	4.84	2.83	2.91	1491
2‐Hexyl‐1‐Decanol	2425‐77‐6	N.D	0.49	N.D	2377
1‐Heptatriacotanol	105794‐58‐9	0.67	0.33	N.D	1678
2‐Butyl‐1‐Octanol	3913‐02‐8	1.17	0.16	N.D	1210
(E)‐2‐Octen‐1‐ol	18409‐17‐1	0.98	N.D	0.40	1616
1‐Octen‐3‐ol	3391‐86‐4	1.19	0.54	0.49	1451
1‐Undecanol	112‐42‐5	N.D	N.D	0.46	1966
1‐Dodecen‐3‐ol	4048‐42‐4	0.10	N.D	0.09	1152
*n*‐Tridecan‐1‐ol	112‐70‐9	N.D	1.02	0.17	1443
Phenol	108‐95‐2	2.89	2.54	1.56	1485
p‐Cresol	106‐44‐5	0.56	0.24	0.18	2085
Pentanal	110‐62‐3	0.51	N.D	N.D	935
Hexanal	66‐25‐1	7.12	4.06	1.42	1083
Heptanal	111‐71‐7	1.19	1.18	0.51	1186
2‐Undecenal	2463‐77‐6	2.04	0.59	0.65	1749
Octanal	124‐13‐0	2.19	1.59	0.52	1289
(E)‐2‐Octenal	2548‐87‐0	1.21	0.52	0.41	1428
Nonanal	124‐19‐6	5.93	4.09	3.16	1394
(E)‐2‐Nonenal	18829‐56‐6	2.30	0.47	0.25	1534
(E)‐2‐Decenal	3913‐81‐3	1.24	0.64	0.77	1642
(E,E)‐2,4‐Decadienal	25152‐84‐5	2.10	0.77	N.D	1807
(E,E)‐2,4‐Dodecadienal	21662‐16‐8	1.14	N.D	N.D	1759
Benzaldehyde	100‐52‐7	1.64	0.93	1.46	1522
Acetophenone	98‐86‐2	N.D	0.57	N.D	1648
Geranylacetone	689‐67‐8	0.66	1.06	0.85	1852
Acetoin	513‐86‐0	8.40	4.80	15.30	1285
2,3‐Pentanedione	600‐14‐6	N.D	N.D	0.23	1061
6‐methyl‐5‐Hepten‐2‐one	110‐93‐0	0.45	1.21	0.60	1338
2‐Nonanone	821‐55‐6	0.09	N.D	0.12	1388
2‐Tridecanone	593‐08‐8	N.D	N.D	0.86	1806
2‐Pentadecanone	2345‐28‐0	1.22	N.D	0.56	2016
2‐pentyl‐Furan	3777‐69‐3	0.51	0.14	0.18	1232
Dibenzofuran	132‐64‐9	0.24	0.16	0.09	2250
Acetic acid	64‐19‐7	N.D	0.52	1.09	1468
4‐Hydroxy‐Butanoic acid	591‐81‐1	N.D	0.57	0.56	1627
Hexanoic acid	142‐62‐1	1.06	N.D	0.56	1855
2‐Ethyl Hexanoic acid	149‐57‐5	0.80	0.26	0.59	1959
Octanoic Acid	124‐07‐2	1.30	0.44	0.59	2070
Cis‐3‐Octyl‐Oxiraneoctanoic acid,	24560‐98‐3	0.53	N.D	N.D	2050
Nonanoic acid	112‐05‐0	1.72	1.16	0.17	2174
*n*‐Decanoic acid	334‐48‐5	3.04	0.88	0.81	2379
Dodecanoic acid	143‐07‐7	1.80	0.74	0.83	2492
3‐Hydroxy‐Dodecanoic acid	1883‐13‐2	0.31	N.D	N.D	1467
Oleic Acid	112‐80‐1	N.D	0.09	N.D	2240
Hexadecanoic acid methyl ester	112‐39‐0	0.52	0.60	0.33	2213
Acetic acid ethenyl ester	108‐05‐4	N.D	N.D	0.48	994
Acetic acid butyl ester	123‐86‐4	0.64	0.29	0.36	1073
Hexanoic acid butyl ester	626‐82‐4	N.D	N.D	0.09	1412
Isopropyl myristate	110‐27‐0	N.D	0.16	0.08	2035
gamma‐Butyrolactone	96‐48‐0	1.29	N.D	N.D	1626
gamma‐Valerolactone	108‐29‐2	0.85	0.41	0.55	1608
5‐Decanolide	705‐86‐2	N.D	N.D	0.17	2184
4‐Dodecanolide	2305‐05‐7	1.55	1.01	N.D	2368
delta‐Dodecalactone	713‐95‐1	0.17	N.D	N.D	2184
Benzothiazole	95‐16‐9	0.42	0.24	0.25	1547
2‐Acetylthiazole	24295‐03‐2	3.48	0.90	1.57	1646
2,3‐Dihydro‐Thiophene	1120‐59‐8	N.D	N.D	0.16	2066
Dimethyl sulfone	67‐71‐0	0.66	0.34	0.20	1900
3‐(Methylthio)‐Propanal	3268‐49‐3	N.D	N.D	0.61	1456
2‐Methyl‐1‐Butanethiol	1878‐18‐8	N.D	0.49	N.D	1436

Note

N.D: Not detected.

The flavor was a very important but complex attribute of mutton sensory quality and was a decisive factor for consumers' judgment before eating. Lipids played an important role in the overall aroma of mutton soup. Many volatile compounds were hydrophobic substances and could facilitate the adsorption and retention by lipids. Most aroma compounds were derived from lipids, such as aldehyde compounds, ester compounds, and acid compounds. If lipids were removed from mutton, it was difficult to distinguish the meat species from the senses. Lipid oxidation was a rather complex process in which hydroperoxides and other primary products were oxidized by polyunsaturated fatty acids. The primary oxidation products were mainly mostly hydroperoxides which show odorless and tasteless and could form a series of secondary oxidation products through further oxidation reaction, which had a strong flavor (Feng et al., 2020) The primary oxidation products were intermediate product of lipid reaction, easy to further reaction, and showed no obvious smelling, had less contribution to the overall flavor. A series of secondary reactions occurred after primary self‐oxidation, leading to the degradation of hydroperoxides forming a wide range of compounds, Most of them were small molecule volatile substances which had strong flavor activity, including thiobarbituric acid reactive substances and volatile compounds, especially volatile aldehydes which had lower threshold, trace concentrations could show significant flavor; therefore, it contributes greatly to the overall flavor.

Lipid oxidation was an important reaction. Alcohols, acids, aliphatic aldehydes, and aliphatic hydrocarbons were detected in the soup. Moreover, C6‐C10 linear chain carboxylic acids in esters were also formed by lipid oxidation or lipolysis probably. Linear aldehydes result from unsaturated fatty acids oxidation and branched aldehydes from amino acid degradation. Oxidation and hydrolysis of unsaturated fatty acids were the main ways of mutton soup flavor formation, heptanal, octanal, nonanal, 2‐decenal, and 2‐nonenal were derived from oleic acid according to lipids oxidative and degradation, and they could provide pleasant aroma (Chen et al., 2019; Zhang et al., 2020). Pentanal, hexanal, (E)‐2‐Octenal, and (E, E)‐2,4‐Decadienal were formed from linoleic acid by oxidation and hydrolysis (Shahidi & Zhong, 2010). Hexanal was a major oxidation product of linoleic acid and other ω‐6 fatty acids. It was the main aldehyde produced in lipid oxidation, which could effectively evaluate the oxidation level of lipids (Muela et al., 2012). Aldehyde compounds were the most typical and interesting lipid‐derived volatiles and significantly affected the product flavor because of their low threshold. They contributed grass, tallow, and woody odor to the overall aroma of mutton soup. The contents of aldehyde compounds showed a significant difference among the three batches, and the PUW batch had the highest content, and the NXW batch showed the lowest content. The result may be related to the ions. The PUW batch had almost no ions, and the state of ultrapure water was very sensitive, and the pH value, ions, and oxygen content change fast. Pentanal was only detected in the PUW batch, which showed fruit and bread aroma. Octanal had the highest content (2.19 μg/ml) and showed the orange, slight grease, and mel aroma derived from auto‐oxidation of oleic acid. Hexanal and nonanal were derived from n‐6 and n‐9 polyunsaturated fatty acid oxidation, respectively, such as linoleic and arachidonic acids. Heptanal showed sweet apricot and nut aroma and was considered as a good indicator of the oxidation level in many meat products (Zamora et al., 2008). (E)‐2‐nonenal, which showed a strong fat aroma, the presence of excess might lead to flavor deterioration. The content of aldehydes associated with alcohols and furans, and aldehydes could inhibit the absorption of alcohols and furans by macromolecules in meat products.

Alcohol compounds usually arose from lipid oxidation (Petričević et al., 2018) and their corresponding aldehyde compounds under oxidation reaction. Generally, alcohols are considered not important to meat flavor because of their high odor thresholds. For example, 1‐octen‐3‐ol was detected in all three batches and had significant contributions to the soup, which showed a strong mushroom aroma. According to the reaction of oxygen with octene group which cracked from hydroperoxide on 12th carbon of arachidonic acid, octenoxyl radical formed firstly, and then further reaction. Acid compounds arose from the corresponding aldehyde compounds oxidation or fatty acids degradation. The direct contribution of acidic compounds to the mutton soup was mild because of its higher threshold, but it also showed an important effect since they were the important substrates for the formation of ester compounds. Branch chain fatty acids were related to "mutton flavor" in the aroma of cooked mutton, such as 4‐methyloctanoic acid, 4‐ethyloctanoic acid, and 4‐methylnononic acid (Wong et al., 1975), but they were not detected in all batches and consistent with the sensory results of mutton soup. The sulfur compound was a special substance and showed a significant effect on the mutton soup, which could provide a special meat flavor. Their thresholds were particularly low but still could play an important role with very low content. Acetophenone, 2‐ethyl‐1‐Hexanol, and 2‐hexyl‐1‐Decanol came from amino acid catabolism. Because of their higher thresholds, hydrocarbons derived from lipids oxidation had little contribution to the mutton soup flavor, but they were also important. The overall flavor of the mutton soup was not decided by one or several substances but by all volatile and nonvolatile compounds.

### 3.5 Analysis of electronic nose

**FIGURE 1:**
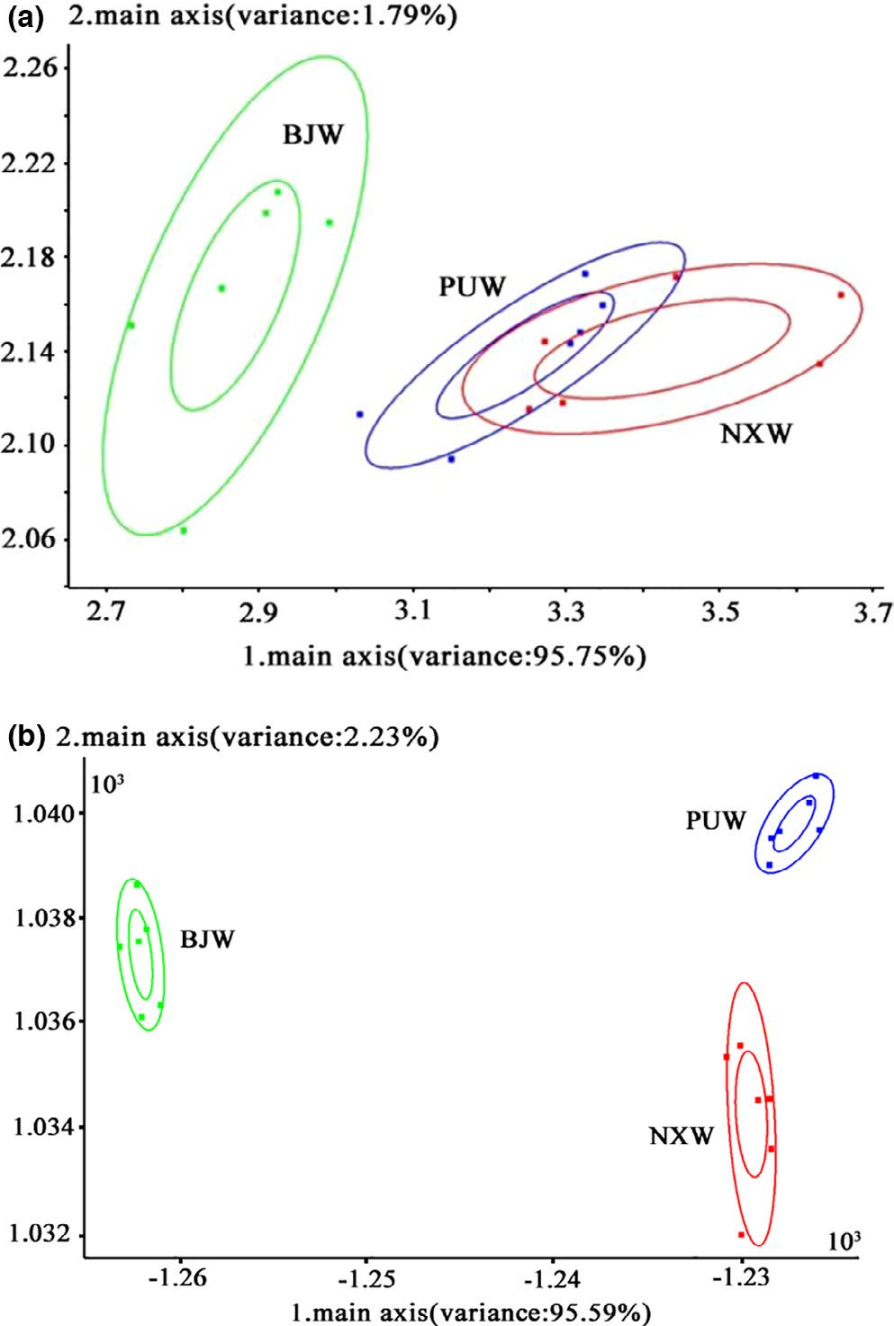
Effect of aroma in different batches of mutton soups with principal component analysis (a) and linear discriminant analysis (b)

The aroma of mutton soups was compared according to Pen3 electric nose, a sensor array of 10 electrodes. The results were responded to 10 electrodes and reduced dimension via PCA and LDA (Fedorov et al., 2021). The effect of aroma in different batches of mutton soups was summarized in Figure 1. Figure 1(a) shows that the main components 1 and 2 contributed 95.75% and 1.79% of the total mutton soups, respectively. Specifically, the aroma of the PUW batches of soup overlapped with the NXW batches of soup using principal component analysis and indicated that the soup cooking with Ningxia water and ultrapure water had a similar aroma but significantly different from BJW batches of soup. A further analysis with linear discriminant analysis and the result was shown in Figure 1(b). The main components 1 and 2 contributed 95.59% and 2.23% respectively, and three batches of mutton soup had no overlapping. LDA was closely related to analysis of variance (ANOVA) and regression analysis and closely related to principal component analysis and factor analysis. So, three batches of mutton soup had significant differences, but the soup cooking with Ningxia water and ultrapure water had a similar aroma, according to the PCA.

### 3.6 Analysis of electronic tongue

**FIGURE 2:**
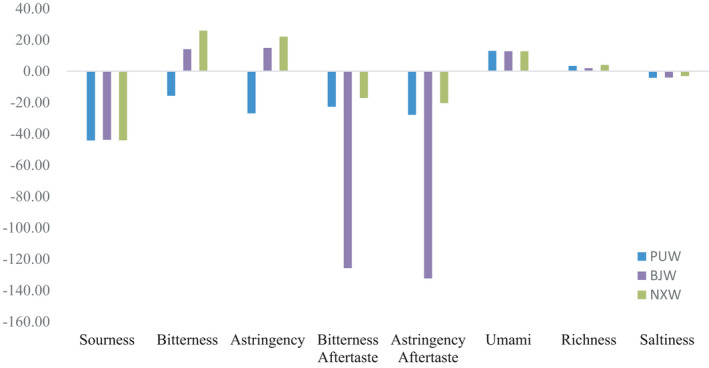
Effect of taste in different batches of mutton soup

The electronic tongue selected taste ingredients based on the electrostatic or hydrophobic interaction between the artificial bilayer lipid membrane on the surface of the sensor and the taste substance and converted the taste perception into numerical form by changing the electric potential, so that quantified the sensor's response (Zhang et al., 2016). The effect of aroma in different batches of mutton soup was summarized in Figure 2 and Table 5. Richness, sourness, and umami taste showed particularly similar during three batches of mutton soup but the bitterness, astringency, and saltiness showed significant differences during three batches, and the result corresponded to the ions in water. Na^+^ often showed saline taste, K^+^ showed bitterness taste, and Mg^2+^ and Ca^2+^ had a certain astringency taste, so that ions had a direct effect on the overall taste.

**TABLE 5:** Differences of taste in different batches of mutton soup

	PUW	NXW	BJW
Sourness	−44.20 ± 0.17	−44.16 ± 0.37	−43.84 ± 0.06
Bitterness	−15.67 ± 12.22^a^	25.88 ± 4.97^c^	14.06 ± 35.36^b^
Astringency	−26.90 ± 13.84^a^	22.02 ± 5.64^c^	14.84 ± 53.28^b^
Bitterness after taste	−22.75 ± 0.64^a^	−17.10 ± 3.21^a^	−125.78 ± 21.83^b^
Astringency aftertaste	−27.85 ± 0.77^a^	−20.35 ± 3.81^a^	−132.42 ± 24.92^b^
Umami	12.91 ± 0.09	12.75 ± 0.05	12.73 ± 0.11
Richness	3.30 ± 0.28^a^	3.96 ± 0.30^a^	1.93 ± 0.42^b^
Saltiness	−4.21 ± 0.05^a^	−3.12 ± 0.06^c^	−4.03 ± 0.09^b^

Note

For the letters a‐c, mean values (*n* = 3) with different letters within the same row are significantly different (*p < .05*).

## 4 CONCLUSIONS

This study showed that different water had significant effects on mutton soup flavor due to the difference of the ions in water, especially Na^+^, K^+^, Ca^2+^, and Mg^2+^, related to the types and concentration. Types and content of free amino acids, nucleotides, and volatile compounds in three batches of mutton soup were different. The results of electronic nose and electronic tongue were also significantly different. The PUW batch had the highest free amino acids and volatile compounds, while the NXW batch had the highest taste nucleotides. Adjusting the concentrations and types of ions in cooking water could alter the content of free amino acids, nucleotides, and volatile compounds and improve the overall flavor of the mutton soup. In order to improve the quality of the mutton soup, it is wisdom to confect appropriate ionic type and content of water.

However, further studies to explore the relationship among lipid oxidation and lipolysis, protein oxidation, and degradation with the ions in water during mutton soup processing were necessary.

## CONFLICTS OF INTEREST

The authors declare that they have no conflicts of interest.

## AUTHOR CONTRIBUTIONS

**Bing zhao:** Data curation (lead); Funding acquisition (lead); Writing‐original draft (lead). **Shouwei Wang:** Conceptualization (lead); Methodology (lead). **Baoguo Sun:** Project administration (lead); Resources (lead). **Yuyu Zhang:** Formal analysis (equal); Methodology (equal); Writing‐review & editing (equal). **Qianrong Wu:** Data curation (equal); Methodology (equal). **Mingwu Zang:** Data curation (equal); Methodology (equal). **Le Wang:** Data curation (equal); Formal analysis (equal); Methodology (equal); Writing‐review & editing (equal). **Hui Wang:** Data curation (equal); Methodology (equal).

## Data Availability

The data used to support the findings of this study are available from the corresponding author upon request.
